# Human iPSC-Derived Blood Vessel Organoids for Studying Chronic Hypoxia-Induced Microvascular Dysfunction

**DOI:** 10.1369/00221554261437861

**Published:** 2026-05-11

**Authors:** Paola Serrano Martinez, Maxime Cammeraat, Amber Teppema, Cornelis J. F. van Noorden, Reinier O. Schlingemann, Ingeborg Klaassen

**Affiliations:** Ocular Angiogenesis Group, Department of Ophthalmology, Amsterdam UMC location University of Amsterdam, Amsterdam, The Netherlands; Microcirculation, Amsterdam Cardiovascular Sciences, Amsterdam, The Netherlands; Ocular Angiogenesis Group, Department of Ophthalmology, Amsterdam UMC location University of Amsterdam, Amsterdam, The Netherlands; Ocular Angiogenesis Group, Department of Ophthalmology, Amsterdam UMC location University of Amsterdam, Amsterdam, The Netherlands; Microcirculation, Amsterdam Cardiovascular Sciences, Amsterdam, The Netherlands; Ocular Angiogenesis Group, Department of Ophthalmology, Amsterdam UMC location University of Amsterdam, Amsterdam, The Netherlands; Ocular Angiogenesis Group, Department of Ophthalmology, Amsterdam UMC location University of Amsterdam, Amsterdam, The Netherlands; Microcirculation, Amsterdam Cardiovascular Sciences, Amsterdam, The Netherlands; Ocular Angiogenesis Group, Department of Ophthalmology, Amsterdam UMC location University of Amsterdam, Amsterdam, The Netherlands; Microcirculation, Amsterdam Cardiovascular Sciences, Amsterdam, The Netherlands

**Keywords:** age-related macular degeneration, capillary organoids, capillary-like structures, diabetic retinopathy, in vitro microvessels, tumor angiogenesis, vascular networks, vascular organoids, vessel networks

## Abstract

Microvascular dysfunction due to hypoxia is a key contributor in the pathogenesis of many disorders including cancer and retinal and cardiovascular diseases, but relevant human models are missing. Here, we present a robust 3D in vitro method with the use of human induced pluripotent stem cell–derived blood vessel organoids to analyze in vitro microvascular remodeling. We present a detailed practical pipeline combining optical tissue clearing, high-resolution immunofluorescence, and surface marker analysis to quantitatively assess hypoxia-driven changes in endothelial cells, pericytes, and the basal lamina. Exposure of these blood vessel organoids to chronic hypoxia (1% O_2_) for 1 week recapitulated key pathological features, including structural remodeling and a dysregulated secretome with altered vascular endothelial growth factor signaling. This approach establishes a versatile and human-relevant platform to study microvascular remodeling induced by chronic hypoxia and other pathological stimuli and their contribution to microvascular-related diseases:

## Introduction

Microvascular dysfunction underlies several life-threatening microvascular-related disorders, such as coronary, cerebral and diabetic microvascular diseases, sepsis, thrombosis, acute respiratory distress syndrome, and cancer.^1^ To fully understand the pathophysiology of microvascular-related disorders, there is a pressing need to develop models that can recapitulate the function of the human microvasculature.

Recent research advancements have led to the generation of blood vessel organoids, which are self-organizing three-dimensional (3D) structures derived from human-induced pluripotent stem cells (iPSCs) that recapitulate key cellular, molecular, and functional features of the human microvasculature.^2–4^ This model has shown potential in mimicking microvascular changes induced by diabetic conditions^4^ and displaying microvascular remodeling induced by metabolic alterations.^5^ Therefore, blood vessel organoids offer a promising tool for disease modeling and drug development; however, the power of this system has still not been fully explored.

Microvascular remodeling and other changes can occur in response to hypoxia, which is a crucial factor involved in the development and progression of several pathological conditions.^1,6–10^ Hypoxia is classified by duration as either acute or chronic.^11^ Whereas the effects of acute hypoxia are often reversible, chronic hypoxia can lead to cell death.^12^ Romeo and colleagues^5^ recently reported that exposing human iPSC-derived blood vessel organoids to acute hypoxia triggers structural remodeling of their capillaries. However, the effect of chronic hypoxia on the blood vessel organoid microvasculature remains to be investigated. A study of chronic hypoxia using blood vessel organoids could reveal the potential of this 3D human model for determining the pathological impact of sustained vascular hypoxia in diseases such as muscular sclerosis,^9^ motor neuron disease,^9^ age-related macular degeneration,^6^ Alzheimer’s disease,^8,10^ chronic kidney disease,^7^ and proliferative diabetic retinopathy.^13^

Here, we demonstrate that human iPSC-derived blood vessel organoids provide a model of the human microvasculature that sustains chronic hypoxia, enabling the study of prolonged hypoxia-induced microvascular remodeling in vitro.

## Material and Methods

### iPSC Maintenance

The human iPSC lines (LUMC0072iCTRL01^14^ and LUMC0065iCTRL08^15^) used in this study were generated by the Leiden University Medical Center (LUMC) iPSC core facility, Leiden, The Netherlands. Detailed information on the iPSC lines can be found in the Human Pluripotent Stem Cell Registry (https://hpscreg.eu/). iPSC lines were cultured on Vitronectin XF (7180, STEMCELL Technologies, Saint-Égrève, France)-coated non-tissue culture-treated plates (27147, STEMCELL Technologies) in mTeSR Plus (100-0276, STEMCELL Technologies) in a humidified incubator at 37C in 5% carbon dioxide (CO_2_).

### Generation of Blood Vessel Organoids

Blood vessel organoids were generated from iPSCs according to the protocol of Wimmer and colleagues.^3,4^ Briefly, for differentiation, 2 × 10^5^ iPSCs per well were resuspended in aggregation medium containing 50 μM of Y-27632 (1254, RayBiotech, Peachtree Corners, GA) and seeded into wells of an ultra-low-adherent six-well plate (38071; STEMCELL Technologies). Aggregates were incubated in a humidified incubator at 37C in 5% CO_2_ for 2 days. Next, aggregates were resuspended in an N2B27 medium containing a neurobasal medium (21103-049; Gibco, Waltham, MA): DMEM/F12 (11330-032, Gibco) 1:1, 0.5% N2 supplement (11520536, Gibco), 1% B27 supplement (11500446, Gibco), 0.5% GlutaMAX (1342629, Gibco), 55 μM β-mercaptoethanol (31350010, Thermo Fisher Scientific, Waltham, MA), 100 U/ml Penicillin-Streptomycin (P/S, 15140122, Gibco) supplemented with 12 μM CHIR99021 (4423, Tocris Bioscience, Bristol, United Kingdom), and 30 ng/ml bone morphogenetic protein 4 (BMP-4; 130-111-165, Miltenyi Biotec, Bergisch Gladbach, Germany) and transferred back to the low-attachment plate to induce mesoderm formation in a humidified incubator at 37C in 5% CO_2_. After 3 days, aggregates were collected by gravitation, resuspended in the N2B27 medium supplemented with 100 ng/ml vascular endothelial growth factor (VEGF, VE5-H4210, ACROBiosystems, Newark, NJ) and 2 μM forskolin (72112, STEMCELL Technologies), and replated into the low-attachment plate to grow in a humidified incubator at 37C in 5% CO_2_. Two days later, aggregates were collected and embedded in collagen type I (PureCol, Bovine Collagen, 42557, CellSystems GmbH, Troisdorf, Germany)-Matrigel (356231, Corning Life Sciences B.V., Amsterdam, The Netherlands) and maintained in StemPro-34 SFM medium containing StemPro-34 nutrient supplement (11580356, Gibco), 1% GlutaMAX, 100 U/ml P/S, 15% fetal bovine serum (FBS; F7524, Merck Life Science, Amsterdam, The Netherlands), 100 ng/ml VEGF, and 100 ng/ml fibroblast growth factor 2 (FGF-2, 139-093-839, Miltenyi Biotec) to induce capillary network formation in a humidified incubator at 37C in 5% CO_2_;. After 5 days, capillary networks were extracted from the collagen type I–Matrigel matrix and either collected for blood vessel organoid formation or processed for immunofluorescence imaging or flow cytometry analysis. For blood vessel self-assembly and maturation, single capillary networks were transferred to individual wells of a PrimeSurface 96U Plate (PrimeSurface 96U-MS-9096UZ; PHC Europe B.V., Breda, The Netherlands), containing 100 μl of StemPro-34 SFM medium, supplemented with StemPro-34 nutrient supplement (11580356, Gibco), 1% GlutaMAX, 100 U/ml P/S, 15% FBS, 100 ng/ml VEGF, and 100 ng/ml FGF-2. After 4 days of culture, another 100 μl of medium was added to the organoids. A full media change was done after 1 week in culture, and after 10 days in culture, 100 μl medium was supplemented to the organoids. Blood vessel organoids were maintained under these conditions for 14 days in a humidified incubator at 37C in 5% CO_2_.

For hypoxia treatment, 7-day-old blood vessel organoids in fresh 100 μl of medium per well (StemPro-34 SFM medium, supplemented with StemPro-34 nutrient supplement, 1% GlutaMAX, 100 U/ml P/S, 15% FBS, 100 ng/ml VEGF, and 100 ng/ml FGF-2) were cultured in 1% oxygen (O_2_) for 1 week in a hypoxia chamber (Whitley H35 hypoxystation, LA Biosystems B.V., Waalwijk, The Netherlands). After 3 days, 100 μl of pre-equilibrated medium (equilibration in the hypoxic chamber 24 hr prior to addition) was supplemented to the organoids.

### Flow Cytometry Analysis of Capillary Networks and Blood Vessel Organoids

A flow cytometry analysis was performed following the protocol of Wimmer et al.,^3,4^ with some small adaptations. Capillary networks were collected, minced using a scalpel, and enzymatically and mechanically digested in phosphate-buffered saline (PBS, 10010015, Gibco) containing 2.5 mg/ml collagenase type I: collagenase type II, 10 mg/ml dispase (16131740, Gibco), and 1 mg/ml DNase-I (11284932001, Roche, Landsmeer, The Netherlands) for 30 min at 37C with shaking in a heat block.

Blood vessel organoids were collected and enzymatically digested in PBS containing 2.5 mg/ml collagenase type I (17100017, Gibco), 2.5 mg/ml collagenase type II (17101015, Gibco), 10 mg/ml dispase, and 1 mg/ml DNase-I. The digestion was carried out for 45 min at 37C with shaking in a heat block.

Single cells derived from the capillary networks or blood vessel organoids were resuspended in a flow cytometry buffer containing PBS with 2% FBS and 2 mM ethylenediaminetetraacetic acid (EDTA, 15825388, Thermo Fisher Scientific) and stained for 45 min at 4C in the dark, using the following antibodies: anti-platelet-derived growth factor receptor beta (PDGFRβ, phycoerythrin (PE), 1:100, CD140b-Clone 28D4, 558821, BD Biosciences, Drachten, The Netherlands), anti-platelet endothelial cell adhesion molecule (PECAM-1 or CD31, fluorescein isothiocyanate (FITC), 1:50, clone WM59, 303103, BioLegend Europe BV, Amsterdam, The Netherlands), anti-CD34 (allophycocyanin (APC), 1:50, QBEND-10, FAB7227A, Bio-Techne, Minneapolis, MN), and anti-VE-cadherin (CD144, BV786, 1:50, 565672, BD Biosciences). 4′,6-Diamidino-2-phenylindole (DAPI; 250 ng/ml, 62248, Thermo Fisher Scientific) staining was used to exclude dead cells. Data was acquired using a BD symphony A1 cell analyzer, and the flow cytometry results were analyzed using FlowJo v10.10.0 Software (BD Life Sciences, Franklin Lakes, NJ). The percentage of double-positive CD31/VE-cadherin endothelial cells (ECs) and PDGFRβ-positive pericytes was used to determine the EC-to-pericyte ratio in capillary networks and blood vessel organoids.

### Enzyme-linked immunosorbent assay

To analyze VEGF levels secreted by cells from the blood vessel organoids (secretome), the conditioned medium was collected from 29-day blood vessel organoids, cultured under normoxia or hypoxia, and stored at −20C until analysis. VEGF levels were measured using a human VEGF quantikine enzyme-linked immunosorbent assay (ELISA) kit (DVE00, R&D Systems, Minneapolis, MN), following the manufacturer’s instructions.

### Fixation of Capillary Networks and Blood Vessel Organoids

Capillary networks were rinsed with Hanks’ balanced salt solution (HBSS, 14065056, Gibco), fixed with 4% formaldehyde (28908, Thermo Fisher Scientific) in HBSS for 15 min at room temperature (RT) and washed three times with HBSS.

Blood vessel organoids were washed with HBSS, fixed with 4% formaldehyde in HBSS for 4 hr at 4C on a rolling platform, and washed three times with HBSS.

For the blood vessel organoids cultured under hypoxia, the first wash and the addition of the fixative was done in the hypoxia chamber. After fixation, the organoids were washed three times with HBSS.

### Whole-Mount Immunostaining of Capillary Networks and Blood Vessel Organoids

Whole-mount immunostaining of capillary networks and blood vessel organoids was performed following the SunJin Lab immunostaining protocol (RapiClear 1.47 datasheet, http://www.sunjinlab.com). All incubations were performed on an orbital shaker.

Fixed capillary networks were incubated for 3 hr at RT, in blocking buffer consisting of 1% bovine serum albumin (BSA, 10735094001, Roche), 1% Triton X-100 (T8787, Sigma-Aldrich, St Louis, MO), 2.5% dimethyl sulfoxide (DMSO, 276855, Sigma-Aldrich), and 0.2% sodium azide (1.06688.0100, Merck Life Science) in PBS. No prior permeabilization was required. Capillary networks were washed three times in PBS for 15 min and stained overnight at 4C using the following primary antibodies resuspended in blocking buffer: anti-CD31, anti-PDGFRβ, anti-CD34, and anti-alpha smooth muscle actin (α-SMA). Stained capillary networks were washed three times for 1 hr each at RT with washing buffer consisting of PBS with 0.05% Tween20 (P1379, Sigma-Aldrich). Afterwards, capillary networks were incubated with the respective fluorescently labeled secondary antibodies, DAPI (250 ng/ml), and/or directly conjugated primary antibodies (Phalloidin Alexa Fluor 488, 1:200, A12379, Thermo Fisher Scientific) in blocking buffer for 3 hr at RT. The following secondary antibodies were used: donkey anti-sheep, donkey anti-goat, donkey anti-mouse (A32773). After secondary antibody (AB) incubation, capillary networks were washed three times for 1 hr each at RT with washing buffer, followed by three 20-min washes in PBS at RT. Before mounting, RapiClear CS mounting gel (RCCS004, SunJin Lab Co., Taiwan, R.O.C) was pre-warmed to 60C and cooled down to 40C. Capillary networks were mounted with RapiClear CS mounting gel in iSpacer (IS206, SunJin Lab Co.) microchambers.

Fixed blood vessel organoids were permeabilized in 2% Triton X-100 in PBS solution containing 0.05% sodium azide for 2–3 days at 35C. Next, blood vessel organoids were washed three times for 15 min each in PBS. They were then blocked in a solution of 10% normal goat serum (0060-01, Southern Biotech, Birmingham, AL), 1% Triton X-100, 2.5% DMSO, and 0.2% sodium azide in PBS, incubating at 4C for 1–2 days. After three washes for 15 min in PBS on an orbital shaker, blood vessel organoids were stained for 3–5 days (first overnight at RT and then at 4C), using the following primary antibodies resuspended in antibody dilution buffer (1% normal goat serum, 0.2% Triton X-100, 2.5% DMSO, and 0.05% sodium azide in PBS): anti-CD31, anti-PDGFRβ, anti-CD34, anti-α-SMA, anti-VE-cadherin, anti-collagen type IV, anti-laminin. Stained blood vessel organoids were washed three times with washing buffer for 1 hr at RT and then kept in washing buffer containing 3% sodium chloride (NaCl, 1064040500, Supelco, St Louis, MO) and 0.2% Triton X-100 in PBS at 4C for 1–2 days. Afterwards, blood vessel organoids were incubated with the respective labeled secondary antibodies, DAPI (1 µg/ml), and/or primary conjugated antibodies (Phalloidin Alexa 488, 1:200, A12379, Thermo Fisher Scientific) in AB dilution buffer for 2–3 days at 4C. The following secondary antibodies were used: donkey anti-sheep, donkey anti-goat, goat anti-mouse (A-11031), donkey anti-mouse (A-31571), goat anti-mouse (115-545-146), or goat anti-rabbit. After secondary antibody incubation, blood vessel organoids were washed three times with washing buffer for 1 hr each at RT and then kept in washing buffer, at 4C, overnight. Next, blood vessel organoids were washed three times with PBS for 20 min each and cleared with a 37C pre-warmed RapiClear CS mounting solution (RCCS001, SunJin Lab Co.), overnight to 24 h at RT, and subsequently mounted with RapiClear CS mounting gel (RCCS004, SunJin Lab Co.) in iSpacer (IS206, SunJin Lab Co.) microchambers. RapiClear CS mounting gel was pre-warmed to 60C and cooled down to 40C before mounting.

Detailed information of the primary and secondary antibodies used for the staining of capillary networks and blood vessel organoids is available in Tables 1 and 2.

**Table 1.** Primary Antibodies.

Antibody	Working Dilution	Identifier	Manufacturer
Anti-CD31	1:50	AF806	R&D systems
Anti-PDGFRβ	1:100	AF385	R&D systems
Anti-CD34	1:100	M0014	Sanquin
Anti-α-SMA	1:100	M0851	Agilent Technologies
Anti-VE-cadherin	1:100	36-1900	Thermo Fisher Scientific
Anti-collagen type IV	1:250	MA122148	Thermo Fisher Scientific
Anti-laminin	1:200	ab11575	Abcam

Sanquin (Amsterdam, The Netherlands), Agilent Technologies (Santa Clara, CA), Abcam (Amsterdam, The Netherlands).

**Table 2.** Secondary Antibodies.

Antibody	Conjugate	Working Dilution	Identifier	Manufacturer
Donkey anti-sheep	Alexa Fluor 647	1:200	A-21448	Thermo Fisher Scientific
Donkey anti-goat	Cy3	1:100	705-165-147	Jackson ImmunoResearch
Donkey anti-mouse	Alexa Fluor Plus 555	1:500	A32773	Thermo Fisher Scientific
Goat anti-mouse	Alexa Fluor 568	1:200	A-11031	Thermo Fisher Scientific
Donkey anti-mouse	Alexa Fluor 647	1:500	A-31571	Thermo Fisher Scientific
Goat anti-mouse	Alexa Fluor 488 AffiniPure	1:200	115-545-146	Jackson ImmunoResearch
Goat anti-rabbit	Alexa Fluor 488	1:200	A-11036	Thermo Fisher Scientific

Jackson ImmunoResearch (West Grove, PA).

Samples were imaged using a Ti2 ZDrive confocal microscope (Nikon Europe BV, Amstelveen, The Netherlands) equipped with a CrestOptics X-Light spinning disk (pinhole 50 µm, CrEST X-Light V3, Nikon Europe BV) or a STELLARIS 8 confocal microscope (Leica Microsystems, Wetzlar, Germany). Acquired images were processed using ImageJ^16^ 1.50i. Time-lapse bright-field imaging was generated using a Incucyte S3 (Sartorius Netherlands B.V., Amersfoort, The Netherlands).

### Statistical Analysis

All experiments were performed at least in triplicate. Data is presented as mean ± standard error of the mean (SEM). Between-session variation from multi-session experiments was removed using Factor Correction version 10.5.^17^ Unpaired *t*-test with Welch’s correction was used to determine statistical significance, with *p*<0.05 considered significant. GraphPad Prism version 10.2.0 for Windows (GraphPad Software, Boston, MA, www.graphpad.com) was used for statistical analysis.

## Results

### Generation of iPSC-Derived Blood Vessel Organoids

**Figure 1.**
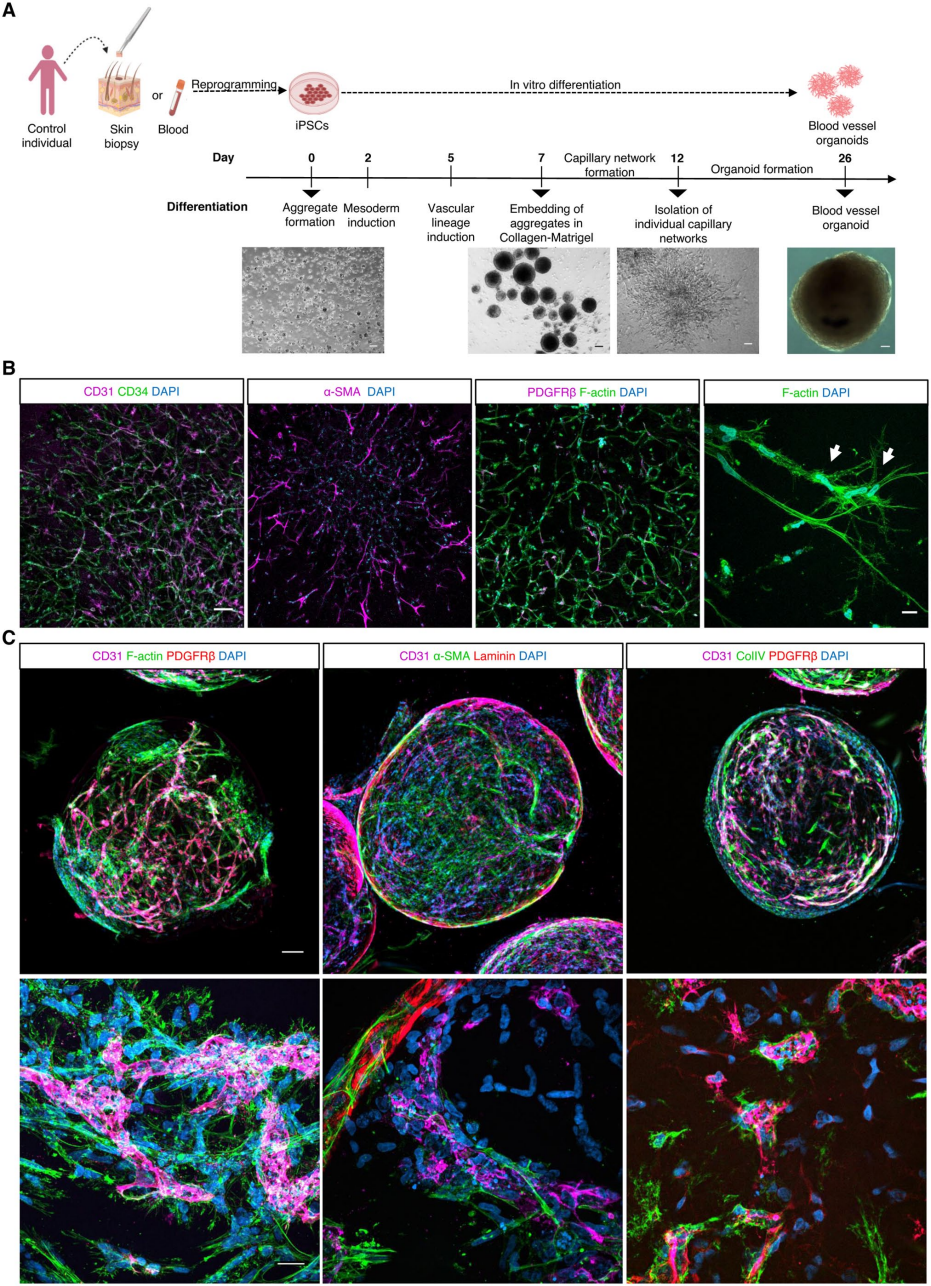
Generation of blood vessel organoids from human iPSC control donors. (A) Schematic overview of the protocol for the generation of capillary networks that self-assemble into blood vessel organoids. The cartoons were created with BioRender.com. Scale bars: 100 μm. (B) Whole-mount immunostaining of phalloidin (F-actin), CD31, CD34 (endothelial markers), PDGFRβ, α-SMA (pericyte markers), and DAPI (nuclei) expression in generated capillary networks (day 12). Scale bar: 100 μm. Cells which morphologically resemble tip cells (white arrowheads) were observed in the angiogenic sprouts of developing capillary networks. Scale bar: 20 μm. (C) Whole-mount immunostaining of blood vessel organoids (day 26) for CD31, phalloidin, laminin and collagen type IV (basal lamina markers), PDGFRβ, and α-SMA (mural cell markers). Scale bars: 100 μm in the upper panel, 20 μm in the lower panel.

**Figure 2.**
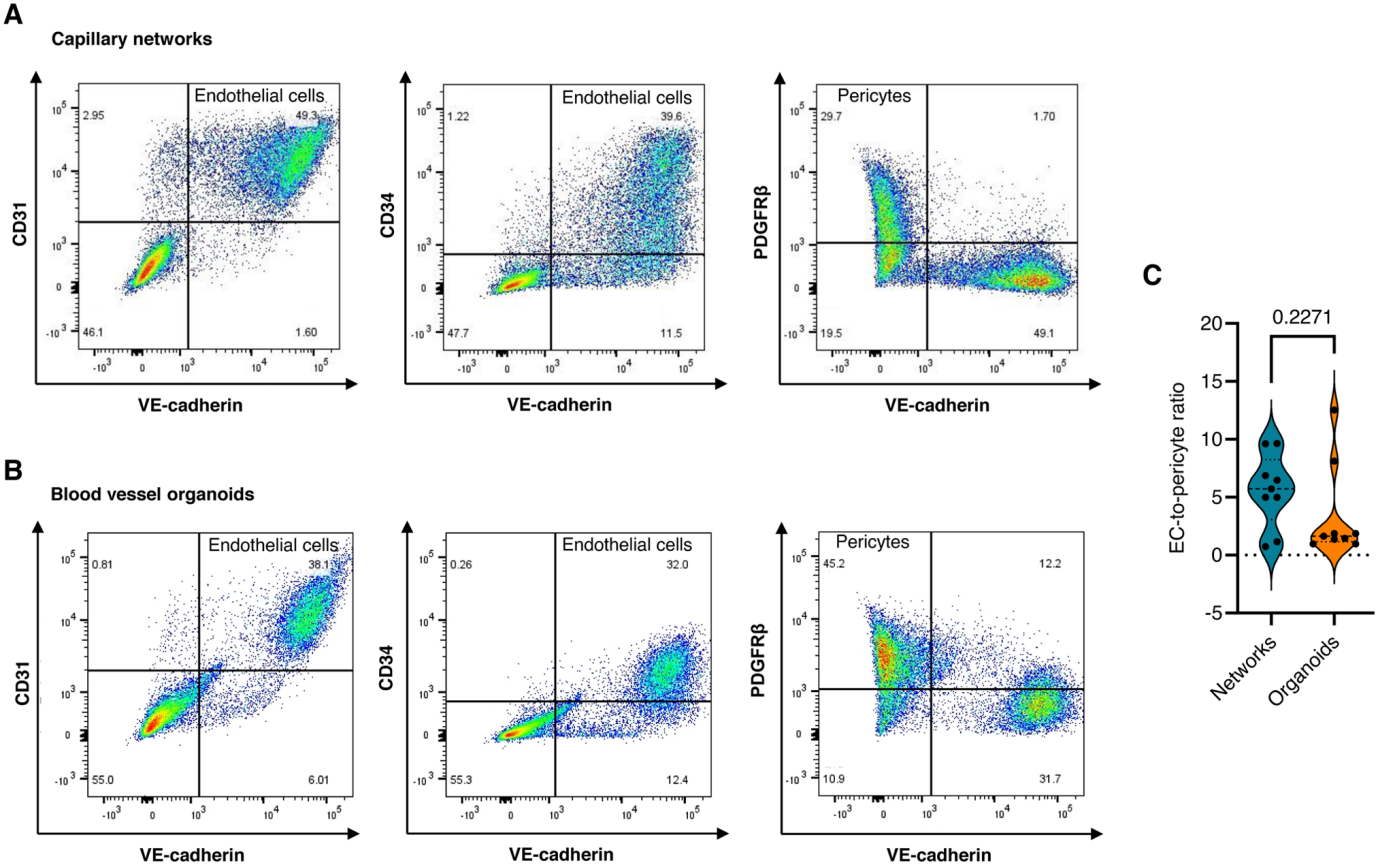
Analysis of the relative amounts of ECs and pericytes in capillary networks and blood vessel organoids. (A) Flow cytometry analysis (presented in scatter plots) was used to determine the different cell populations in capillary networks analysis (day 12). (B) The presence of endothelial and mural cells in the blood vessel organoids (day 26) was confirmed by flow cytometry analysis (presented in scatter plots). (C) The EC-to-pericyte ratio in capillary networks (day 12) and blood vessel organoids (day 26), based on the percentage of double-positive CD31/VE-cadherin ECs and PDGFRβ-positive pericytes as determined by flow cytometry analysis. All values are represented as mean ± SEM, *p*<0.05.

We generated capillary networks that self-assemble into 3D blood vessel organoids from healthy control human iPSCs. During the differentiation process, iPSCs first differentiated into capillary networks, which further matured into blood vessel organoids over 26 days (Fig. 1A). Immunofluorescence analysis of these capillary networks showed the presence of CD31- and CD34-expressing ECs, as well as PDGFRβ^+^ and α-SMA^+^ pericytes (Fig. 1B). The presence of endothelial markers, such as CD31, CD34, and VE-cadherin, and the pericyte marker PDGFRβ was further confirmed by flow cytometry analysis (Fig. 2A). In addition, in the angiogenic sprouts of developing capillary networks, cells that morphologically resemble tip cells^18^ were observed, as shown by F-actin staining (Fig. 1B). Following extraction from Matrigel, the single-capillary networks self-organized into blood vessel organoids (Supplemental Video S1). These organoids contained a mesh of lumenized vessels formed by CD31/VE-cadherin/CD34-positive ECs and PDGFRβ/α-SMA-positive pericytes, enveloped by a collagen type IV–containing basal lamina (Fig. 1C). Flow cytometry analysis further confirmed the expression of the endothelial markers CD31, VE-cadherin, and CD34, as well as the pericyte marker PDGFRβ (Fig. 2B). Further analysis of the flow cytometry data indicated that the self-assembly of capillary networks into blood vessel organoids was accompanied by a decrease in the EC-to-pericyte ratio (Fig. 2C).

### Exposure of Blood Vessel Organoids to Chronic Hypoxia

**Figure 3.**
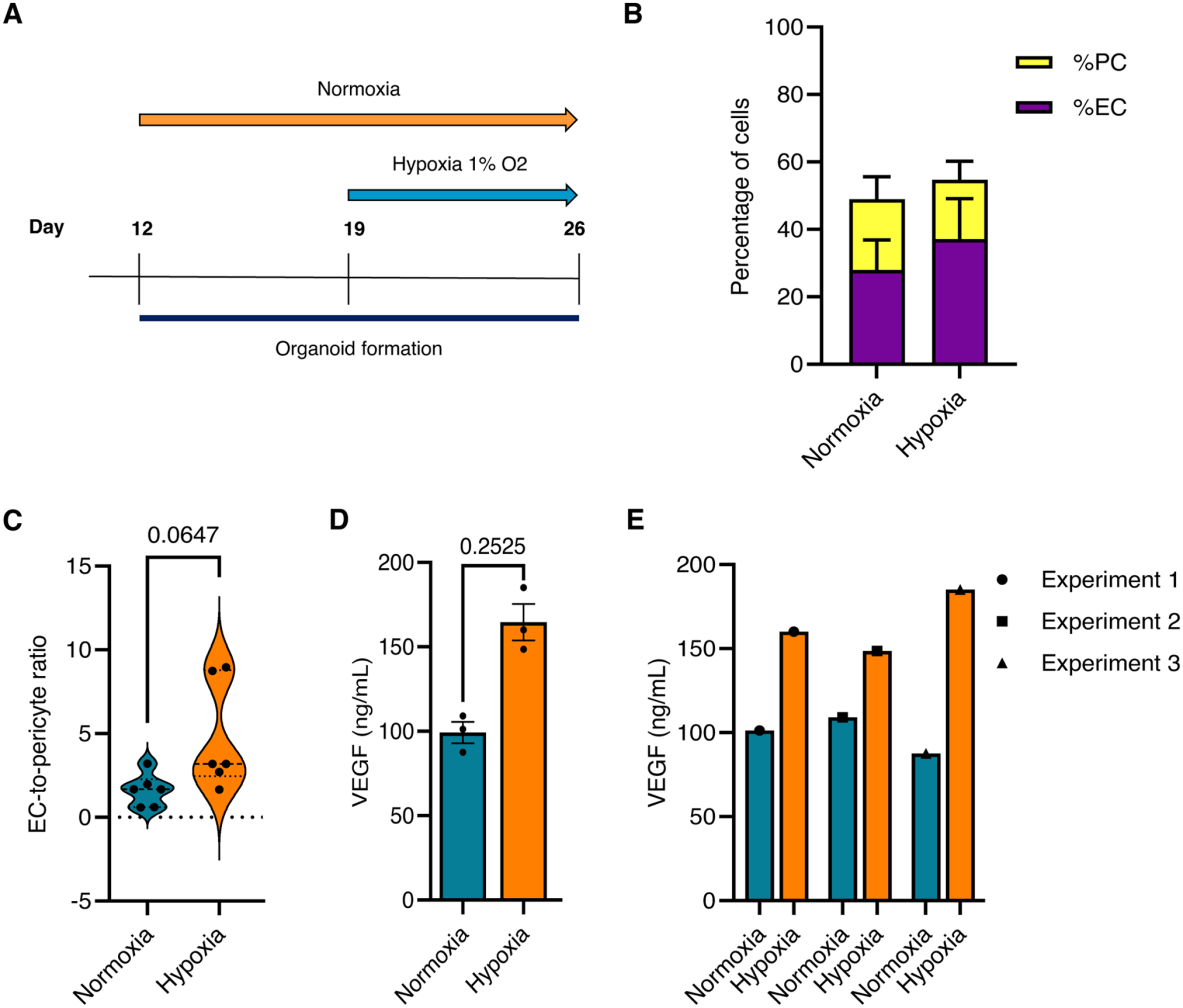
Exposure of blood vessel organoids to chronic hypoxia. (A) Schematic timeline of blood vessel organoid exposure to normoxia (~20% O_2_) or hypoxia (1% O_2_). (B) Percentage of double-positive CD31/VE-cadherin ECs and PDGFRβ-positive pericytes in blood vessel organoids (day 26) treated with normoxia and hypoxia, measured with flow cytometry analysis. (C) Assessment of the EC-to-pericyte ratio, based on the percentage of CD31^+^/VE-cadherin^+^ ECs and PDGFRβ^+^ pericytes determined by flow cytometry analysis, in blood vessel organoids (day 26) treated with normoxia and hypoxia. In organoids under hypoxia, the EC-to-pericyte ratio increased as compared to normoxia. (D) VEGF secreted levels in conditioned medium from blood vessel organoids (day 26) under chronic hypoxia significantly increased as compared to normoxia (quantified by ELISA), which as reproduced in (E) three consecutive independent experiments. All values are presented as mean ± SEM, *p*<0.05.

**Figure 4.**
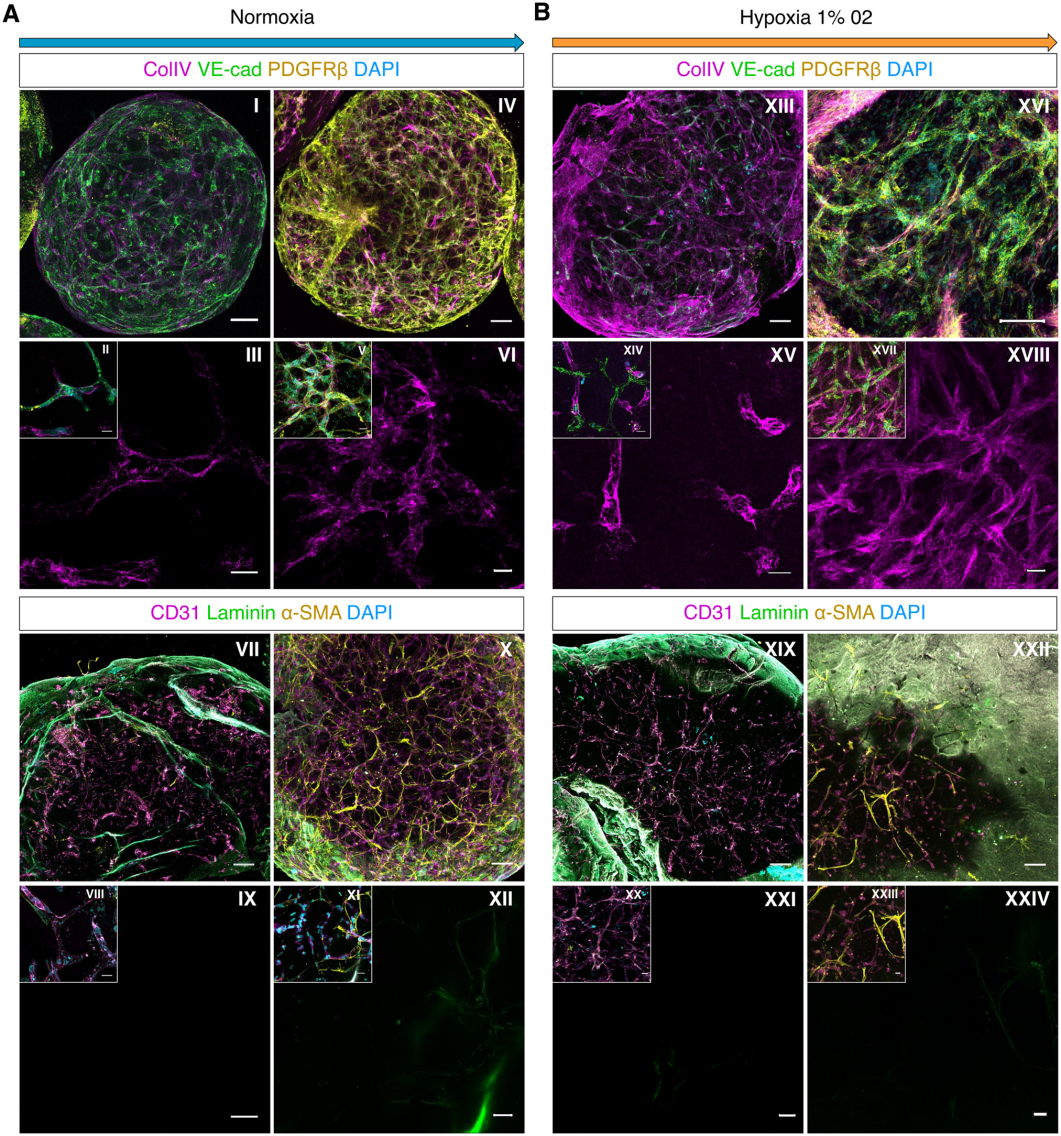
Whole-mount confocal imaging of blood vessel organoids cultures under normoxic and chronic hypoxic conditions. (A) In normoxia (day 26), blood vessel organoids formed mature 3D blood vessels consisting of VE-cadherin-positive ECs, α-SMA- and PDGFRβ-positive pericytes, as well as a basal lamina indicated by the expression of collagen type IV (ColIV) and laminin (I-XII). (B) Blood vessel organoids under hypoxia (day 26) developed a matured vascular network (XIII-XXIV) with increased collagen type IV deposition (XIII-XVIII). The deposition of laminin was not increased after exposure of the organoids to chronic hypoxia (XIX-XXIV). I, IV, VII, X, XIII, XVI, XIX XXII visualization of whole organoids, scale bars: 100 μm. I, VIII, XI, XIV, XVII, XX, XXIII magnification of the vasculature in the middle of the organoid, scale bars: 20 μm. III, VI, XV, XVIII magnification of the vasculature in the middle of the organoid showing only CollV expression, scale bars: 20 μm. IX, XII, XXI, XXIV magnification of the vasculature in the middle of the organoid showing only laminin expression, scale bars: 20 μm.

To investigate the effects of chronic hypoxia on microvascular integrity, we cultured blood vessel organoids in 1% O_2_ for 1 week (Fig. 3A). Flow cytometry analysis showed an increase in the percentage of ECs and a reduction in the percentage of PDGFRβ-positive pericytes in blood vessel organoids grown under chronic hypoxia (Fig. 3B), resulting in a higher EC-to-pericyte ratio as compared to those grown under normoxic conditions (~20% O_2_) (Fig. 3C). Furthermore, VEGF secretion into the conditioned medium was significantly increased in chronically hypoxic organoids compared to normoxic controls, a result that was consistent across three consecutive experiments (Fig. 3D and E). Microscopy imaging further revealed that the blood vessel organoids under chronic hypoxia developed a vascular network with increased collagen type IV deposition (Fig. 4A I-VI, B XIII-XVIII). The deposition of laminin was not increased in blood vessel organoids cultured under hypoxic conditions compared to organoids grown under normoxia (Fig. 4A VII-XII, B XIX-XXIV).

## Discussion

Hypoxia is a crucial factor in the pathogenesis of several diseases and can drive microvascular remodeling. It can be classified as either acute or chronic. Whereas the effects of acute hypoxia are often reversible, chronic hypoxia can cause permanent deleterious changes. To fully understand the molecular and cellular mechanisms underlying chronic hypoxia-related microvascular disorders, it is necessary to develop novel in vitro models that accurately mimic the human microvasculature. This is particularly important considering the limited availability of human vascular tissue and the physiological differences, high cost, and limited translatability of animal models. Although microvascular dysfunction triggered by acute hypoxia has previously been simulated in blood vessel organoids,^5^ modeling the effects of chronic hypoxia in a complex human microvascular system has remained unexplored.

Here, we investigated the use of human iPSC-derived blood vessel organoids as a proof-of-principle 3D model to study microvascular changes induced by chronic hypoxia. We demonstrate that these organoids tolerate chronic hypoxia (1% O_2_ for 1 week), exhibiting significant microvasculature and secretome alterations, thereby providing a relevant 3D human model to study hypoxia-induced microvascular remodeling in disease.

In this study, we generated blood vessel organoids that recapitulate key structural aspects of the human microvasculature, including ECs, pericytes, and a basal lamina.^1,19^ This finding aligns with and builds upon the work of Wimmer et al.,^3,4^ confirming the robustness of this model. However, we did not use their immunofluorescence protocol^3,4^ to image our capillary networks and blood vessel organoids, as it caused light scattering and poor visualization of internal structures. To enable high-resolution 3D imaging of both models, we introduced optical clearing^20,21^ into the whole-mount immunofluorescence protocol, a modification that was crucial for the successful visualization of our generated capillary networks and blood vessel organoids.

Wimmer and colleagues have previously described that during the generation of blood vessel organoids, capillary networks are initially formed by the sprouting of vessels and further mature to establish a stable vasculature.^3^ The confirmation of such observations in our study allows us to define these two distinct stages as separate in vitro models. Model 1, capillary networks, a model of active angiogenesis, is characterized by vessel sprouting guided by tip cells. Model 2, blood vessel organoids, is a model of a more mature, stable vasculature. Although these structures have been used independently in perturbation studies, either as capillary networks^2^ or blood vessel organoids,^4,5^ their formal distinction as separate models system suitable for different study questions has not been previously described. In our present study, we employed Model 2, the blood vessel organoid model, to study the effects of chronic hypoxia on a stabilized vascular system as a proof of principle.

Romeo et al.^5^ studied microvascular remodeling in human iPSC-derived blood vessel organoids under 1% O_2_ for 24 hr, a condition defined as acute hypoxia.^22^ This treatment resulted in reduced pericyte coverage, vessel length, and vessel density compared to normoxic controls. In our model, chronic hypoxia (1% O_2_ for 1 week) led to an increase in the EC-to-pericyte ratio. Of note, we use the term “hypoxia” to refer to experimental hypoxia, in which the O_2_ level is determined relative to the level found in a cell culture incubator, independent of the O_2_ level to which cells are exposed to in vivo.^23^ Our observation of an increased EC-to-pericyte ratio, suggesting a drop in the relative amount of pericytes, was similar to that observed in acute hypoxic conditions.^5^ In vivo, despite variations in pericyte coverage among tissues, its alteration can lead to vascular disturbances regardless of the tissue type.^24,25^ Moreover, the change in the EC-to-pericyte ratio in our model following chronic hypoxia was accompanied by increased VEGF secretion and increased collagen deposition without a corresponding increase in laminin. These overall results are consistent with microvascular remodeling during angiogenesis. In vivo, angiogenesis can be triggered by tissue injury. Such damage results in hypoxia and the concomitant transcriptional activation of VEGF by hypoxia-inducible factors (HIFs).^26,27^ Consequently, the release of VEGF stimulates ECs and activates pericytes to orchestrate angiogenesis.^28^ Vascular stability is then achieved through the deposition of a vascular basal lamina, representing the final step in angiogenesis.^29^ Angiogenesis can occur under physiological conditions, such as in wound healing, or in a pathological manner. Whereas pericyte proliferation takes place during wound healing,^28,30–33^ pericyte loss has been reported in some forms of pathological angiogenesis.^34–39^ Therefore, our findings suggest that chronic hypoxia is achieved in our model, and it drives pathological angiogenesis, characterized by VEGF release, a concomitant increase in the EC-to-pericyte ratio, and the deposition of collagen type IV. Nevertheless, it remains to be confirmed whether true angiogenesis occurred in our study and whether the observed decrease in the EC-to-pericyte ratio is due to either a true loss of pericytes, a change in pericyte marker expression, or proliferation of ECs. Furthermore, hypoxia may induce collagen type IV and laminin deposition in a context-dependent and isoform-specific manner^40,41^ which may explain our observed differences in the deposition of collagen type IV and laminin. However, we cannot rule out that the injury induced by chronic hypoxic exposure led to an aberrant deposition of the basal lamina, a process associated with fibrosis.^42^ Another challenge remains in recapitulating tissue-specific heterogeneity of the endothelium^43,44^ within the blood vessel organoids and O_2_ requirements associated with the tissue of interest.^45^ Therefore, future work should aim to generate tissue-specific blood vessel organoids by driving cellular specification with different growth factors or through co-culture with tissue-specific cells. Subsequently, the applied O_2_ level could be adjusted to match that of the endothelium of interest, physiological hypoxia, or tailored to its specific metabolic requirements, cellular hypoxia.^23^

Overall, our approach of exposing blood vessel organoids to chronic hypoxia provides a novel model for studying pathological angiogenesis and offers new insights into the cellular mechanisms of vascular remodeling in disease. Furthermore, the potential to use this model with organoids derived from patients with conditions like proliferative diabetic retinopathy and age-related macular degeneration may help elucidate the currently unknown pathophysiology of these diseases.
